# Restricted nutrient intake does not alter serum-mediated measures of implant response in cell culture

**DOI:** 10.1186/2049-1891-4-45

**Published:** 2013-11-19

**Authors:** Christopher D Reinhardt, Tiffany L Lee, Daniel U Thomson, Laman K Mamedova, Barry J Bradford

**Affiliations:** 1 Department of Animal Sciences and Industry, 232 Weber Hall, Kansas State University, Manhattan, KS 66506, USA; 2 Department of Clinical Sciences, A-111 Mosier Hall, Kansas State University, Manhattan, KS 66506, USA

**Keywords:** Beef cattle, Implant, Muscle, Myosin heavy chain, Nutrient restriction, Satellite cells

## Abstract

**Background:**

During nutritional stress, reduced intake may reduce the efficacy of anabolic implants. This study was conducted to evaluate basic cellular responses to a growth promotant implant at two intake levels.

**Methods:**

Sixteen crossbred steers (293 ± 19.3 kg) were used to evaluate the impact of anabolic implants in either an adequate or a restricted nutritional state. Steers were trained to individual Calan gates, and then randomly assigned to 1 of 4 treatments in a 2 × 2 factorial arrangement. Treatments consisted of: presence or absence of an anabolic growth implant (Revalor-XS, 200 mg TBA and 40 mg estradiol; IMPLANT or CONTROL) and a moderate energy, pelleted, starting cattle diet fed at either 2.0 × or 1.0 × maintenance energy (NE_M_) requirements (HIGH or LOW). Serum (d 0, 14, and 28) was used for application to bovine muscle satellite cells. After treatment with the serum (20% of total media) from the trial cattle, the satellite cells were incubated for 72 h. Protein abundance of myosin heavy chain (MHC), phosphorylated extracellular signal-related kinase (phospho-ERK), and phosphorylated mammalian target of rapamycin (phospho-mTOR) were analyzed to determine the effects of implant, intake, and their interaction (applied via the serum).

**Results:**

Intake had no effect on MHC (*P* = 0.85) but IMPLANT increased (*P* < 0.01) MHC abundance vs. CONTROL. Implant status, intake status, and the interaction had no effect on the abundance of phospho-ERK (*P* ≥ 0.23). Implanting increased phospho-mTOR (*P* < 0.01) but there was no effect (*P* ≥ 0.51) of intake or intake × implant.

**Conclusions:**

The nearly complete lack of interaction between implant and nutritional status indicates that the signaling molecules measured herein respond to implants and nutritional status independently. Furthermore, results suggest that the muscle hypertrophic effects of anabolic implants may not be mediated by circulating IGF-1.

## Background

Anabolic implants increase average daily gains 15 to 20%, and improve feed efficiency by 6 to 14% in feedlot cattle [1]. However, when animals first arrive in a feedlot many are in a state of stress and have not yet become accustomed to the environment, feeding routine, other animals, and other factors, especially if they have traveled long distances or have recently been weaned. These cattle are considered at high risk to develop bovine respiratory disease (BRD). Many of these high-risk cattle have reduced intakes for up to three weeks after arrival at the feedlot [2].

Stress and infection can lead to a hypermetabolic state, characterized by weight loss, increased resting metabolic rate, and muscle protein catabolism [3]. In cattle infected with BRD, decreased feed intake, weight loss, and decreased body condition are often observed [3]. Although ruminants may have a greater capacity to store digesta because of their larger digestive tracts, this weight loss and decrease in body condition may be comparable to the hypermetabolic state that develops in non-ruminants [3]. Munson et al. [4] reported that delaying implant administration in high-risk calves did not alter feedlot performance or the risk of respiratory disease, suggesting that stress brought on by the transition to the feedlot did not diminish the anabolic signals from implanting early in the finishing phase.

Anabolic implants impact growth rate at least in part by stimulating muscle hypertrophy [5]. Androgenic and estrogenic steroids increase *in vitro* bovine satellite cell proliferation, and these effects are mediated in part by the extracellular signal-regulated kinase (ERK) and phosphatidylinositide 3-kinase/AKT/mammalian target of rapamycin (mTOR) signaling pathways [6]. Activation of these pathways, however, is dependent on adequate nutritional status, which is sensed through a complex array of signals [7,8]. Therefore, animals in a depleted nutritional state, accompanied by alterations in hormonal signals, may not have the expected muscle growth response to anabolic implants.

The objectives of this study were to examine the effects of anabolic implants on measures of nutrient balance, metabolic status, and growth factors in animals consuming nutrients either adequate or inadequate to support growth. Moreover, effects of serum-derived signals on *in vitro* proliferation of bovine muscle satellite cells were also assessed.

## Materials and methods

### Animals

All procedures conducted in the present study were approved by the Kansas State University Institutional Animal Care and Use Committee (Protocols 2745.0 and 2745.1).

Sixteen crossbred steers (293 ± 19.3 kg) were purchased locally and transported to Kansas State University. They were weighed, tagged, and vaccinated with a 5-way respiratory viral vaccine and a 7-way clostridial vaccine. The steers were trained to individual Calan® gates over a period of 4 wk.

After training was complete, the steers were randomly assigned to 1 of 4 treatments in a 2 × 2 factorial arrangement. Main effect treatments were as follows: administration or absence of an anabolic implant (Revalor-XS, 200 mg TBA and 40 mg estradiol 17-β [Merck Animal Health, Whitehouse Station, NJ]; IMPLANT or CONTROL, respectively) and either 2.0 × or 1.0 × maintenance energy (NE_M_) requirements of a moderate energy, pelleted, starting cattle diet (HIGH or LOW, respectively; Table 1). Energy content of the diet was calculated from ingredient composition and tabular estimates of individual ingredient energy values [9].

**Table 1:** Ingredient and nutrient composition (dry matter basis) of the diet fed during the experimental period

Ingredient, %	
Alfalfa meal	33.00
Corn gluten feed, dry	18.18
Wheat middlings	14.63
Distillers grains, dried	11.52
Cracked corn	10.94
Cottonseed hulls	7.75
Molasses, liquid	2.00
Limestone	1.85
Salt	0.10
Bovatec 91^1^	0.025
Vitamin A 650	0.006
Zn SO_4_	0.0055
Nutrient composition (calculated)^2^	
CP, %	15.31
Ca, %	0.56
P, %	1.43
NE_M_, Mcal/kg	1.44
NE_G_, Mcal/kg	0.85

^1^The final diet contained 49.89 mg lasalocid sodium per kg.

^2^Nutrient composition calculated based on ingredient composition in the final diet using tabular estimates of nutrient values (NRC, 1996).

Steers were weighed on d 0, 14, and 28 of the trial. At each of these time points, maintenance energy requirements were calculated for each steer [10], and the diet was fed based these calculations and the treatment assignment for each animal.

### Biochemical assays

All assays were conducted in duplicate. Satellite cell isolation was conducted as described by Johnson et al. [5]. Using sterile techniques, 500 g of the semimembranosus muscle was dissected from 2 steers which were not part of the present study (18 mo of age). After removal of connective tissue, the muscle was passed through a sterile meat grinder. The ground muscle was incubated with 0.1% pronase in Earl’s Balanced Salt Solution (EBSS) for 1 h at 37°C with frequent mixing. After incubation the mixture was centrifuged at 1,500 × *g* for 5 min, the pellet was suspended in phosphate buffered saline (PBS; Ca- and Mg-free PBS; Life Technologies, Grand Island, NY), and the suspension was centrifuged at 500 × *g* for 10 min. The supernatant was centrifuged at 1,500 × *g* for 10 min to pellet the mononucleated cells. The PBS wash and differential centrifugation were repeated 2 more times. Cell viability was over 90%. The resulting mononucleated cell preparation was suspended, pooled (density 3 × 10^4^ cells/mL) in cold DMEM containing 10% fetal bovine serum (FBS) and 10% dimethylsulfoxide and frozen in liquid nitrogen. Cells were counted using a hemocytometer and 0.4% Trypan Blue solution.

Blood samples (serum and plasma) were collected from each animal at d 0, 14, and 28 of the trial and used in determining levels of total IGF-1, and additional serum samples were collected from each animal for application to satellite cells [11-13] which had been previously isolated. Total IGF-1 concentration was determined in plasma samples using a commercial ELISA kit (Octeia IGF-1, catalog #AC27F1, Immunodiagnostic Systems Inc., Scottsdale, AZ). The kit includes reagents for pre-treatment of samples to release IGF-1 from binding proteins [14]. Frozen satellite cells were re-suspended in DMEM supplemented with 5% FBS and 1% penicillin-streptomycin mixture (containing 10,000 IU/mL penicillin and 10,000 μg/mL streptomycin). Cells were passaged once, then at 70% confluence, cells were incubated in DMEM for 72 h with serum (20% of total media) taken from individual animals on d 0, 14, or 28 and with an additional control containing 20% FBS and no serum from trial cattle. Halting incubation at 72 h prevents over-confluency of the cells, which can lead to excessive differentiation; since these are skeletal muscle cells and would contract or twitch after differentiation, excessive differentiation would lead the cell layers to come off the cell culture plate. All media contained 1% penicillin-streptomycin mixture, but FBS was not added to wells containing treatment serum.

Protein abundance of myosin heavy chain (MHC; d 0, 14, and 28), phosphorylated ERK (phospho-ERK; d 0 and 28), and phosphorylated mTOR (phospho-mTOR; d 0 and 28) was analyzed in differentiated satellite cells to determine the effects of implant, intake, and their interaction (applied via the serum). MHC was used as a marker of myotube formation, and phospho-ERK and phospho-mTOR are regulators of cell proliferation.

### Western blot analysis

Cultured cells were lysed at 4°C with RIPA lysis buffer (50 mmol/L Tris–HCl, pH 7.5, 150 mmol/L NaCl, 1% NP-40, 0.5% sodium deoxycholate, and 0.% SDS; Santa Cruz Biotechnology, Santa Cruz, CA) containing X1 broad-spectrum protease inhibitor cocktail (Protease inhibitor cocktail I; Calbiochem, Gibbstown, NJ) and centrifuged at 15,000 × *g* for 10 min at 4°C. Protein concentration was measured using a Coomassie protein assay kit (Pierce, Rockford, IL) according to Bradford [15]. The proteins in the supernatant were denatured with SDS buffer containing dithiothreitol for 5 min at 95°C. Forty micrograms of protein were loaded in each lane, and proteins were separated using SDS-PAGE on a 4 – 12% TRIS-glycine gel, then transferred to a nitrocellulose membrane (iBlot; Invitrogen, Carlsbad, CA). Membranes were blocked in Tris buffer (pH 7.4) with 5% dry milk powder for 2 h at room temperature and then incubated with the following antibodies: a rabbit anti-MYH (H-300) polyclonal antibody raised against amino acids 1641–1940 of myosin heavy chain 3 (sc-20641; Santa Cruz Biotechnology, Santa Cruz, CA); rabbit anti-phospho-mTOR (Ser 2481) polyclonal antibody (Cell Signaling Technology, Beverly, MA), and rabbit anti-phospho-ERK (Tyr 204; sc-7976, Santa Cruz Biotechnology) overnight at 4°C. All primary antibodies were diluted 1:1,000. After washing, membranes were incubated for 1 h at room temperature with a secondary antibody (anti-rabbit IgG, sc-2301; Santa Cruz Biotechnology) diluted 10,000-fold in Tris buffer (pH 7.4). Immunodetection was performed by chemiluminescence (West-Dura; Thermo Scientific, Waltham, MA), and band images were visualized using a photodocumentation system (ChemiDoc-It Imaging System; UVP Inc., Upland, CA). Densitometry analysis was carried out using Image J (http://rsbweb.nih.gov/ij/).

### Statistical analysis

Animal was the experimental unit for all measures. Data were analyzed using the MIXED procedure of SAS® (v. 9.3; SAS Institute, Cary, NC); IGF-1 and MHC were analyzed using repeated measures and d 0 values were included in the model as a covariate; phospho-ERK and phospho-mTOR were analyzed as a completely randomized design but included d 0 values as a covariate. The independent variables included the main and interactive effects of implant status and intake level. Effects were considered significant with a protected F-test with α < 0.05; effects were considered a trend if *P* < 0.10.

## Results and discussion

Level of feeding achieved different growth rates (0.76 vs. 0.02 kg/d for 2.0 × vs. 1.0 × maintenance intake, respectively; *P* < 0.01); implant status did not affect 28-d growth rates (0.36 vs. 0.41 kg/d for implanted vs. control, respectively; *P* = 0.38). There was no interaction between level of intake and implant status for growth rate (*P =* 0.38).

**Figure 1:**
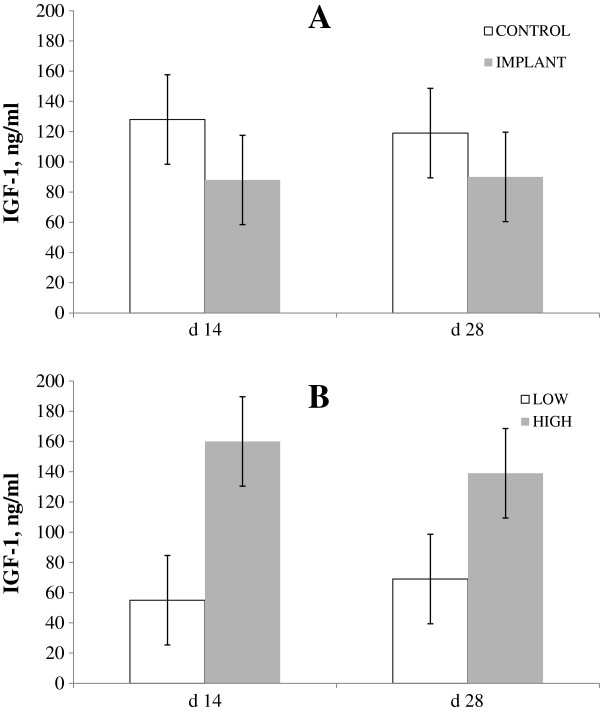
**Effects of implant or intake level on plasma urea nitrogen.** Plasma urea nitrogen concentration of steers (Chart **A**) either implanted (solid line) or not implanted (dashed line) with a long-acting TBA:E_2_ implant; and (Chart **B**) fed a common diet at either 2.0 (solid line; HIGH) or 1.0 (dashed line; LOW) × maintenance NE requirements. (Effect of intake *P* = 0.56; implant *P* = 0.62; intake × implant *P* = 0.99; day × intake *P* < 0.01; day × implant *P* = 0.33; day × intake × implant *P* = 0.12. Pooled SE for treatment means was 0.261. ^a,b^means without a common superscript differ (*P* < 0.05).

There was no intake × implant for serum IGF-1 levels (*P* = 0.56). High-intake steers had greater IGF-1 levels (*P* = 0.02) vs. low-intake steers (Figure 1). Elsasser et al. [16] reported that greater energy intake increases circulating IGF-1 levels in growing steers, and Hayden et al. [17] reported reduced IGF-1 values in steers when intake was restricted by 23%.

The IGF-1 concentrations in implanted steers were not different from those in control cattle in this study. Bryant et al. [18,19] reported no effects of implants on IGF-1 in feedlot heifers until d 42 after implant administration; however, Johnson et al. [18,19] and Reinhardt [20] reported that circulating IGF-1increased in implanted steers by d 21 and d 28, respectively, compared to non-implanted steers. Anderson et al. [21] reported that exogenous estradiol supplementation in post-menopausal women increased circulating growth hormone 2.1-fold but actually decreased circulating IGF-1 by 27%.

**Figure 2:**
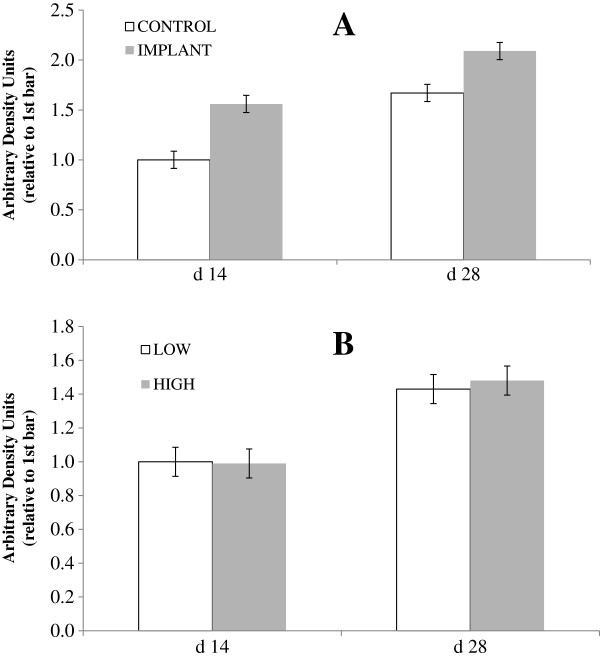
**Effects of implant and intake level on myosin heavy chain abundance in cultured myocytes.** Myocytes from a non-study steer were treated with serum (20% of total media) from study steers drawn on d 0, 14, and 28, and myosin heavy chain abundance was measured. In chart **A**, steers were either implanted (gray bars) or not implanted (open bars) with a long-acting TBA:E_2_ implant; in chart **B**, steers were fed a common diet at either 2.0 (gray bars; HIGH) or 1.0 (white bars; LOW) × maintenance NE requirements. (Effect of day *P* < 0.01; intake *P* = 0.85; implant *P* < 0.01; intake × implant *P* = 0.23; day × intake *P* = 0.88; day × implant *P* < 0.01; day × intake × implant *P* = 0.44; Error bars represent pooled SEM for treatment means = 0.103).

When serum was applied to the cultured satellite cells from the semimembranosus muscle of non-study steers, level of intake had no effect (*P* = 0.85; Figure 2) on MHC abundance but implant increased MHC abundance (*P* < 0.01), with no interaction observed between intake level and implant status (*P* = 0.23). In this study, MHC was used as a marker of myotube formation, reflecting satellite cell proliferation and differentation. Baxa et al. [22] found that implanting increased MHC type IIa mRNA *in vivo*. In a smaller study, Baxa [23] found that implanting with a combined estradiol-17β (E_2_)/trenbolone acetate (TBA) led to numerical increases in MHC type I and IIa mRNA. Chung et al. [24] found no effect of E_2_/TBA implants on MHC type I, IIa or IIx mRNA, but did find that MHC IIa increased over time which was also seen in the present study (effect of day *P* < 0.01). Implanting with E_2_/TBA increases muscle fiber diameter [25,26], due to an initial increase in DNA transcription followed by an increase in nuclei within the muscle fiber which support hypertrophy, but ultimately due to an increase in total protein produced by the cell [27], of which MHC is a key constituent.

**Figure 3:**
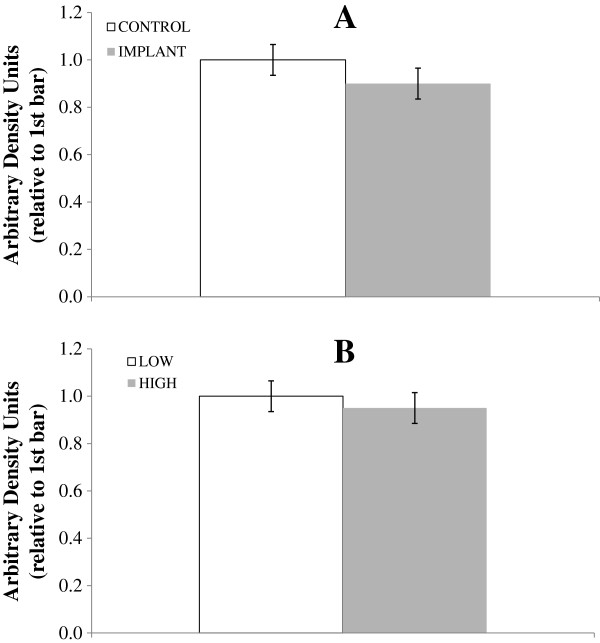
**Effect of implant and intake level on phosphorylated extracellular signal-related kinases (phosphor-ERK).** Myocytes from a non-study steer were treated with serum (20% of total media) from study steers drawn on d 0 and 28, and phospho-ERK abundance was measured. Steers were (Chart **A**) either implanted (gray bars) or not implanted (white bars) with a long-acting TBA:E_2_ implant and (Chart **B**) fed a common diet at either 2.0 (gray bars; HIGH) or 1.0 × (white bars; LOW) maintenance NE requirements. (Effect of intake *P* = 0.65; implant *P* = 0.47; intake × implant *P* = 0.41). Pooled SE for treatment means was 0.065.

Extracellular signal-regulated kinase plays a role in regulating insulin- and potentially IGF-I-mediated glucose uptake by skeletal muscle cells [28], and certain fatty acids reduce muscle cell proliferation via ERK [29]. Implant status did not affect the phospho-ERK abundance (*P* = 0.46; Figure 3). Intake had no effect (*P* = 0.65) on phospho-ERK, and there was no interaction between the two main effects (*P* = 0.41). Because ERK is phosphorylated (activated) by IGF-I signaling [6,30], the lack of phospho-ERK response to implant in the present study is understandable, given the lack of effect on circulating IGF-1. However, intake level also failed to influence phospho-ERK despite the fact that HIGH increased serum IGF-1 compared to LOW.

**Figure 4:**
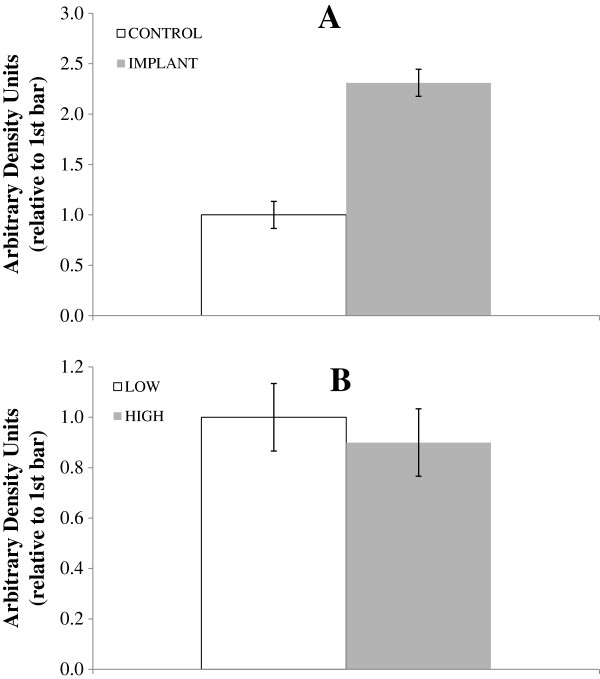
**Effects of implant and intake level on phosphorylated mammalian target of rapamycin (phospho-mTOR) abundance in cultured myocytes.** Myocytes from a non-study steer were treated with serum (20% of total media) from study steers drawn on d 0 and 28. In chart **A**, steers were either implanted (gray bars; IMPLANT) or not implanted (open bars; CONTROL) with a long-acting TBA:E_2_ implant; in chart **B**, steers were fed a common diet at either 2.0 (gray bars; HIGH) or 1.0 (white bars; LOW) × maintenance NE requirements. (Effect of intake *P* = 0.51; implant *P* < 0.01; intake × implant *P* = 0.88. Error bars represent pooled SEM = 0.134).

The protein kinase mTOR plays an essential part in transcription, translation, muscle cell size, and protein stability [31,32]. To help regulate these processes, mTOR integrates information about nutrient status, energy status, and many growth factors and may be a central component in the regulation of cellular processes that determine cell growth and proliferation, and even ultimate overall animal size [33]. Phosphorylation of mTOR can occur at both Ser 2448 [34] and Ser 2481 [35]. We investigated Ser 2481 phosphorylation of mTOR. This site is responsive to nutrient supply and Ser 2481 phosphorylation status is directly correlated with activity of mTOR complex 1, which is responsible for promoting protein synthesis through activation of S6 kinase 1 and eukaryotic translation initiation factor 4E-binding protein 1 [35]. There was no interaction between intake and implant status (*P* = 0.88; Figure 4) for phospho-mTOR, and intake had no effect (*P* = 0.51); however, serum from implanted steers (*P* < 0.01) stimulated greater phospho-mTOR levels vs. serum from CONTROL steers. Phosphorylated mTOR normally increases when nutrition is adequate for growth and decreases when the nutrient environment is not favorable for growth [31,36]. Although we did not see this response, it is important to recognize that our experimental design only partially subjected satellite cells to the milleu that muscle tissue of LOW animals would have seen. Our protocol provided cells with essentially unlimited nutrient supply via the DMEM that comprised 80% of the media, thereby negating effects of intake level on nutrient supply to the satellite cells. The lack of intake effect on phospho-mTOR, therefore, suggests only that endocrine signals (or compounds not supplied in DMEM) that differed by intake failed to alter satellite cell mTOR signaling. Yang et al. [32] reported that mTOR is highly sensitive to upregulation by IGF-1 and that the stress caused by low nutrient availability can inhibit mTOR signaling. In the present study, although we saw no effect of implant on IGF-1, we did measure a strong response of implant on phospho-mTOR. We saw a significant effect of increased intake level on IGF-1 but no corresponding effect of high nutrient intake stimulating phospho-mTOR.

Johnson et al. [18,19] reported increased mitogenic activity of serum from implanted vs. control steers when applied directly to satellite cell cultures, but that study also reported increased levels of circulating IGF-I in the implanted steers. Results from the present study suggest that elevated IGF-I is not the sole serum-mediated factor which increases signalling for increased mitogenic activity *in vitro*, given that serum from nutrient abundant and nutrient restricted calves, which differed greatly in IGF-I concentration, did not alter MHC or phospho-mTOR. The phospho-mTOR and MHC response to implant, in the absence of a circulating IGF-I response, could be explained by a number of additional, non-somatomedin-mediated pathways associated with growth regulation. Binding proteins specific for growth hormone and some, although not all, for IGF-I increase in plasma in response to elevated nutrient availability independent of treatment with exogenous growth hormone [37], and the same study indicated that elevated nutrient status actually reduced growth hormone in plasma. Concentration of IGF-I mRNA is elevated in longissimus muscle cells from animals previously administered exogenous E_2_[38,39], and muscle mRNA concentrations for the adipogenic regulators PPARγ and stearoyl CoA desaturase are reduced after administration of exogenous TBA [24]. In addition, both E_2_ and TBA, which were used in combination in the present study, stimulate protein synthesis and reduce protein degradation in a dose-dependent manner when directly applied to fused satellite cell cultures [40,41]. This action is at least partially modulated through stimulation of muscle IGF-I mRNA by E_2_ and TBA [42].

Treatment of proliferating bovine satellite cells cultures with either E_2_ or TBA resulted in increases in IGF-1 mRNA as well as increased ^3^H-thymidine incorporation rate [42]. Direct treatment of fused bovine satellite cell cultures with E_2_ increases protein synthesis and decreases protein degradation rates, and blocking of estradiol binding suppresses estradiol-induced modifications in protein synthesis and degradation [40]. Testosterone also increases satellite cell activation in rats [43]. These documented effects indicate a direct mechanism for both estradiol and TBA on satellite cell proliferation and protein synthesis.

Implanting with a combination of E_2_ and TBA increases circulating estradiol and TBA, but only exogenous E_2_ increases circulating growth hormone [44-46]. Circulating estradiol is correlated to circulating growth hormone in humans [47], and exogenous E_2_ supplementation increased growth hormone secretion in post-menopausal women [48] and ovariectamized ewes [49]. Sotiropoulos et al. [50] reported that growth hormone stimulates myoblast fusion with myotubes. Ge et al. [51] reported that growth hormone did not stimulate myoblast fusion or proliferation, but growth hormone did increase protein synthesis.

Hepatocyte growth factor increases satellite cell activation [52-54] and fibroblast growth factor enhances proliferation of satellite cells in culture [55,56]. Although hepatocyte growth factor [57-59] and fibroblast growth factor [60,61] are elevated *in vitro* in response to treatment with E_2_, liver and muscle mRNA for hepatocyte growth factor are not affected *in vivo* after implanting cattle with E_2_ and TBA [62].

The satellite cell results, where serum from implanted cattle activated mTOR signaling, make it tempting to speculate that mTOR is a more critical mediator of muscle hypertrophy in response to implants than ERK. Certainly, mTOR has been shown to be a key regulator of protein synthesis [8] and is activated by testosterone treatment [63]. The primary upstream regulators of mTOR are AMP-activated protein kinase (AMPK), AKT, and ERK. Blockade of either the AKT and ERK signalling pathways ablated the mitogenic response to trenbalone acetate in bovine satellite cells, suggesting that at least basal activation of both of these pathways is required [6]. Conversely, activation of AMPK, which is triggered by nutrient deficiency, impairs mTOR phosphorylation , and this pathway has been implicated in loss of muscle mass in nutrient-restricted cows [36]. Given that implant status did not enhance phospho-ERK abundance in this study, we propose that basal ERK signaling was likely necessary to permit activation of mTOR, but that activation of the AKT pathway was probably more directly involved in stimulating mTOR signaling. This is also consistent with the unique roles of ERK and mTOR signaling pathways. Although ERK does contribute to mTOR activation, it is probably more important as a regulator of cell proliferation [64]. Proliferation of satellite cells is important for maintainance of future hypertrophic potential *in vivo*, but myotube formation is driven by cell differentation and activation of protein synthesis, which is more closely related to the function of mTOR.

## Conclusions

No interactions were found between nutrient restriction and implant status in muscle satellite cell culture treated with serum from calves either implanted or not implanted and fed at either 1× or 2 × maintenance. This suggests that the effects of exogenous anabolic agents and nutrient status on growth regulation, at least insofar as it is regulated by the molecules measured in this study, are independent of one another. Serum from implanted cattle promoted increased abundance of the active phospho-mTOR regulatory protein as well as myotube formation, measured by MHC abundance, in bovine satellite cells. These finding suggest the presence of circulating factors in implanted cattle that influence cell signals and promote hypertrophy of muscle tissue.

## Competing interests

The authors declare that they have no competing interests.

## Authors’ contributions

CR is the corresponding author; TL gathered all samples, participated in laboratory procedures, conducted statistical analysis, and wrote the primary draft of the manuscript; DT created the concept and design of the study; LM coordinated all laboratory analyses; BB was responsible for determining the appropriate analytical methods to be conducted. All authors have read and approved the final manuscript.
